# Pharmacological profiling in CLL patients during pirtobrutinib therapy and disease progression

**DOI:** 10.21203/rs.3.rs-6249480/v1

**Published:** 2025-03-31

**Authors:** Varsha Gandhi, Shady Tantawy, Burcu Aslan, Ganiraju Manyam, Lakesla Iles, Natalia Timofeeva, Neetu Singh, Nitin Jain, Alessandra Ferrajoli, Philip Thompson, Keyur Patel, Sai Prasad Desikan, William Wierda

## Abstract

Pirtobrutinib is a reversible Bruton’s tyrosine kinase (BTK) inhibitor that has shown efficacy for patients with chronic lymphocytic leukemia (CLL) in BRUIN trial. These patients were previously treated with covalent BTK inhibitor (cBTKi) and either discontinued cBTKi or had disease progression during therapy. As a result, some patients had wild-type BTK while others had mutant BTK (mostly C481 site where cBTKi binds). All patients received pirtobrutinib monotherapy. Twenty-six patients with CLL from BRUIN were treated at MD Anderson and twenty-three were followed up for at least two years. We compared baseline features between patients who had progressive-disease versus those who remained on therapy during the first 24 cycles of pirtobrutinib therapy. We performed pharmacological profiling of peripheral blood mononuclear cells taken from patients at pretreatment, during pirtobrutinib therapy, and at progression. Relapsed/refractory CLL to prior cBTKi, baseline BTK mutations, unmutated IGHV, bulky lymph nodes, XPO1 mutation and complex karyotype were more prevalent attributes in the pirtobrutinib progressive-disease subgroup. Interestingly, among patients who had progressive-disease, only three patients had baseline wild-type BTK, while eleven had mutant BTK (mostly C481). As reported before, we also observed that C481S mutant clone was decreased during therapy while T474 mutant either developed or increased. We did pharmacological profiling in samples taken during pirtobrutinib therapy when disease is responsive and primary cells are sensitive to pirtobrutinib. We also analyzed sensitivity of CLL cells to other targeted and clinically available agents when patient had PD on pirtobrutinib and needed a new treatment regimen. Ex vivo pharmacologic profiling suggested that during pirtobrutinib therapy, peripheral blood mononuclear cells (CLL cells) became resensitized to ibrutinib and other targeted agents. Combination therapy, including ibrutinib and venetoclax, was effective regardless of genomic background and even after relapse from pirtobrutinib monotherapy.

### Introduction

The B cell receptor (BCR) signaling pathway is integral to survival, migration, proliferation, and maturation of normal B cells and chronic lymphocytic leukemia (CLL) cells (1, 2). Bruton's tyrosine kinase (BTK) is proximal in the BCR signaling pathway and is activated by antigen ligation (3)..

Covalent BTK inhibitors (cBTKi) such as ibrutinib, acalabrutinib, and zanubrutinib are US Food and Drug Administration approved agents that irreversibly bind to cysteine 481 residue in BTK and block enzyme activity and downstream signal transduction (4–7). These drugs have transformed treatment of B-cell malignancies in general and CLL in particular (8). However, there are two main limitations. First, 10–25% of patients with CLL discontinue therapy due to intolerance to these cBTKi. Second, with long-term usage, progressive disease (PD) and resistance can be observed (9). Generally, disease progression was due to somatic mutations in *BTK* (70–80%) or the immediate downstream signaling molecule *PLCG2* (10–13). Within *BTK*, two types of alterations were commonly detected: first mutations are in the kinase domain at the C481 binding site and second variations are gate-keeper mutations in the T474 residue cBTKi alone and combined with other agents are becoming preferred therapeutic options globally and especially in Western world. Resistance to cBTKi is generally due to mutations in the BTK (mostly C481) or PLCG2. This led to the development of ncBTKis that do not require binding to the C481 residue with potential efficacy in patients relapsed to cBTKi. (14–25).

Pirtobrutinib is a ncBTKi that inhibits signaling both from wildtype and C481 mutant *BTK* and was investigated in preclinical (23, 25) and clinical (24, 26) settings. Pirtobrutinib was highly selective for BTK when tested against 370 different kinases (25). In the clinical trial for patients with B-cell malignancies, the drug was well-tolerated with clinical responses in CLL, mantle-cell lymphoma, and Waldenström macroglobulinemia (24, 26–29). Importantly, pirtobrutinib was equally effective in patients who were intolerant to cBTKi as well as those who developed resistance to these cBTKi (23, 30). Consistent with this observation, there were responses in patients with CLL who had wildtype or C481S mutant *BTK* (26). At the molecular level, pirtobrutinib therapy inhibited the BCR pathway, and expression of genes associated with proliferation and immune pathways in both wildtype *BTK* and mutant *BTK* CLL lymphocytes and resistance developed due to non-C481 *BTK* mutations (29, 30).

A recent publication of the multi-center phase I/II pirtobrutinib (BRUIN) trial reported on results for 247 patients with CLL or small lymphocytic lymphoma (SLL) who were previously treated with a cBTKi. At a median follow up of 19.4 months, overall response rate to pirtobrutinib was 82% and 79% for patients who received either cBTKi alone or a combination of cBTKi and Bcl-2i, respectively. The median progression-free survival (PFS) for these two groups was 19.6 and 16.8 months, indicating that many patients have PD within first 2 years of initiating pirtobrutinib (26). Three critical questions emerge from these observations. First, what are determining factors that dictate maintained response versus PD. Second, does treatment with pirtobrutinib exert selective pressure on the population of CLL cells to allow re-sensitization to cBTKi or other targeted agents or combinations. Third, what are potential therapeutic intensification options for patients who have PD on pirtobrutinib, including those who resolve C481S but develop new second site mutant clones, and those who have Bcl-2i resistant disease.

To address these questions, we analyzed data from CLL patients on BRUIN trial treated at MD Anderson Cancer Center (MD Anderson). We compared response to therapy during first two years of pirtobrutinib and associated baseline genetic and genomic features. We did pharmacological profiling in samples taken during pirtobrutinib therapy when disease is responsive and primary cells are sensitive to pirtobrutinib. Finally, we also analyzed sensitivity of CLL cells to other targeted and clinically available agents when patient had PD on pirtobrutinib and needed a new treatment regimen. Our data indicated that Prior cBTKi r/r and baseline mutant BTK was associated with a shorter PFS as compared to prior cBTKi intolerance and baseline WT BTK. Pirtobrutinib therapy can re-sensitize the cells to the action or ibrutinib, and venetoclax combination appears to be an effective approach for those who progressed to pirtobrutinib.

## Patients and Methods

#### *Clinical protocol*

All patients included in current study were participants in the BRUIN trial which is an open-label, multicenter phase 1/2 study of pirtobrutinib in patients with B-cell malignancies (NCT03740529; clinicaltrials.gov). Among 41 patients treated at MD Anderson, 26 were with CLL **(Figure 1A)**. All patients were previously treated with cBTKi and were either intolerant or had relapsed/refractory (R/R) CLL **(Table 1)**. Except for one, all patients received 200 mg/day of pirtobrutinib. Patients provided written informedconsent to participate in the clinical trial and a consent to participate in the accompanying laboratory correlative studies. The clinical trial wasapproved by the MD Anderson Cancer Center's Institutional Review Board and was conducted in accordance with the Declaration of Helsinki.

#### *Clinical laboratory end points*

All patient samples were analyzed genomically using EndCLL or EndLymphoma panel next-generation sequencing (NGS) **(Supplemental Table 1)**. EndLymphoma assay is NGS-based analysis for the detection of somatic mutations in 162 genes related to lymphoma, myeloma and other hematologic neoplasms. Briefly, sequencing libraries were prepared from genomic DNA using hybridization capture-based target enrichment of the genomic regions of interest. Bidirectional paired-end sequencing was performed using NGS platform to screen for single nucleotide variants and small insertions/deletions. The genomic reference sequence used is genome GRCh37/hg19. Covered genes/exons/codons are defined as those having total coverage depth of greater than or equal to 250 reads. A minimum of 10% tumor cells are required in the sample for mutation analysis. We determined the effective lower limit of detection of this assay (analytical sensitivity) to be at 5% (one mutant allele in the background of nineteen wild type alleles) by taking into consideration the depth of coverage at a given base/s.

Determination of *IGHV* gene usage and mutation status and ZAP-70 analyses were performed as described(31). Conventional karyotyping and fluorescence in situ hybridization (FISH) wereperformed by the clinical cytogenetics laboratory, Departmentof Hematopathology at MD Anderson (32). Briefly, karyotyping was performed on metaphase cells prepared from bone marrow (BM) aspirate specimens cultured for 72 hours with mitogen (Pokeweed, Phorbol ester, and oligo nucleotide) using standard techniques. Twenty Giemsa-banded metaphases were analyzed and the results were reported using the International System for Human Cytogenetic Nomenclature (ISCN 2020). FISH for common abnormalities associated with CLL was performed on interphase nuclei obtained from cultured blood or BM cells using a multi-color probe panel designed to detect deletions of 11q22.3 (*ATM*), 13q14.3 (*D13S319*), 13q34 (*LAMP1*), 17p13.1 (*TP53*) and trisomy 12 (*12p11.1-q11*) according to the manufacturer's instructions (Abbott Molecular, Abbott Park, IL). Here are the cut-off values for common abnormalities, you can decide whether you want to include them in your manuscript. 5.1% for *ATM* deletion; 4.5% for *TP53* deletion; 2.4% for trisomy 12; 3.5% for one copy deletion of D13S319 and 0.7% for loss of two copies of D13S319.

#### *Peripheral blood collection and processing of samples*

Samples were collected into heparinized vacutainer (green-top) blood collection tubes. Plasma samples were obtained by centrifugation of blood samples and were stored in aliquots. Cells were isolated using a Ficoll-Hypaque gradient (Atlanta Biologicals, Norcross, GA). The number of cells and cell volume were determined using a Coulter Channelizer (Beckman Coulter, Brea, CA). Cells were used fresh for *ex vivo* drug testing or saved as pellets for immunoblots; WBC and lymphocyte % is provided in each graph and indicate high WBC counts with a high percentage of lymphocytes. Blood samples were collected from patients prior to initiating pirtobrutinib (C1D1), after 1 week (C1D8), 4 weeks or 1 cycle (C2D1), 3 cycles (C4D1), additional cycles (C10-C20) and at time of progression of disease (PD).

#### *Measurement of chemokine levels, cell death, immunoblot assays*

Samples were taken at C1D1 (pretreatment), C1D8, C2D1, C4D1, and at time of PD. In some cases, generally due to closure of laboratory during COVID pandemic, some timepoints are missing. Analysis for CCL3/MIP-1 alpha and CCL4/MIP-1 beta was done using ELISA Kit **(Supplemental Table 3)**. The quantitation was done from the standard curve and the results are presented as the means of technical triplicates in pg/mL.

#### *Ex Vivo apoptosis and viability assays*

Ibrutinib and trametinib were from Selleckchem, venetoclax and APR-246 were from MedChem Express, and AZD5991 was from Chemitech. Pirtobrutinib was provided by Loxo Oncology (Stamford, CT). Stock solutions of all drugs were in DMSO. Time-matched, DMSO-treated cells were used as controls. After incubation with drug(s), cells were harvested and stained with annexin V-FITC (BD Bioscience, Franklin Lakes, New Jersey) and then with propidium iodide (PI) (sigma Aldrich, Burlington, MA). When tested for apoptosis in CD19 and CD5 positive population, data were very similar to whole population. Data were analyzed in Flowjo version 8. Cell viability was also determined using resazurin dye and fluorescence was assessed in the microplate reader. Data were normalized to the DMSO control.

#### *Immunoblot analysis*

Cells were collected during pirtobrutinib therapy and processed as described previously1. Immunoblots were performed with use of cellular protein extracts, and nitrocellulose membranes were probed with indicated antibodies **(Supplemental Table 2)** and visualized with an Odyssey Infrared Imaging System (LI-COR Biosciences, Lincoln, NE) or chemiluminescence.

#### *Statistical analysis*

Fishers exact test was used to determine association between disease status and biomarkers of interest. False discovery rate (FDR) was estimated for multiple testing using Benjamini-Hochberg (BH) procedure. Unless specified otherwise, all graphs (including oncoprint) were prepared using GraphPad Prism software. *P* values $\leq$0.05 were considered statistically significant.

#### *Ethics approval and consent to participate*

All methods were performed in accordance with the relevant guidelines and regulations. Live vertebrates were not used in this project. Patients with CLL disease participated in this project. Collection and use of patient samples were obtained by informed consent from each participant and protocol to collect patient sample was approved by the University of Texas MD Anderson Cancer Center Institutional Review Board.

#### *Data availability and sharing*

Data can be obtained six months after publication by contacting corresponding author who will provide anonymized data for the purpose of conducting additional scientific research.

### Results

#### *Baseline features and patient outcomes on pirtobrutinib*

All patients treated at MD Anderson were included and monitored for response, toxicity, and progression. Median PFS was 18.5 months **(Supplemental Figure 1).** Among the 26 patients with R/R CLL who had previously received cBTKi, 23 were available for these investigations and were categorized as having PD within £ 24 cycles versus those with PFS > 24 cycles on pirtobrutinib treatment **(Table 1)**. Among the 23 patients, 14 patients (61%) had PD within first two years **(Figure 1A)**. The 9 patients still on therapy have received 30-42 cycles and are continuing pirtobrutinib therapy (**Supplemental Table 3**). Age, gender, ECOG performance status and number of prior therapies did not correlate with PD, although there were higher number of patients (64%) in the PD group who had received Bcl-2i **(Figure 1B)**. We compared *BTK* and *PLCG2* mutational landscape at baseline. Among the 14 patients who progressed, one had *PLCG2* mutation at baseline, three patients had WT *BTK* at the baseline; and 11 patients had baseline C481 *BTK* mutation **(Figure 1C).** Majority of C481 *BTK* variants were C481S or C481R. Two patients also had gatekeeper T474I alteration. At disease progression, two patients-maintained WT *BTK*. Five patients had development of L528W clone. Expansion or origination of *BTK* T474 clones were observed in several patients while *BTK* C481S variant was either reduced or eliminated **(Figure 1C)**. We further summarized baseline genomics and cytogenetic features illustrating them by oncoprint **(Figure 1D)**. Among 15 attributes evaluated, there were 6 features that associated with PD with an FDR threshold of 15%. Although total number of patients is small and need to be validated in larger cohort, a few observations were clear. All patients who had PD were R/R to prior cBTKi while patients on therapy were both R/R and intolerant to cBTKi (p = 0.014). Consistent with this observation, 80% patients with PD had baseline *BTK* mutations. Comparatively, only 22% with baseline *BTK* mutations are still on therapy (p = 0.013). Patients in the PD subgroup showed association with some poor prognostic features such as presence of bulky lymph nodes (p = 0.048), and higher prevalence (75%) of complex karyotype, CK (p = 0.020). Although all patients who progressed had bassline unmutated IGHV and those with baseline XPO1 mutation (n=5) developed PD, they did not reach statistical significance and (p=0.057 for both of them) and need to be confirmed on larger patients cohort. In contrast, *TP53* or *ATM* aberrations were distributed among both cohorts. Collectively, those patients with early (£ 24 cycles pirtobrutinib) PD

were more likely to have progression on prior cBTKi, baseline *BTK* mutation and complex karyotype. Those who completed >24 cycles of pirtobrutinib were more likely to have discontinued prior cBTKi due to intolerance, have baseline wildtype-*BTK*, and mutated *IGHV* **(Figure 1D).**

***Association of baseline features with progressive disease after pirtobrutinib therapy***

There were 5 features that were significantly associated with progression free survival in this cohort. First, rates of progression during pirtobrutinib therapy in patients who had R/R disease on prior cBTKi were 37% and 53% at 12- and 18-months, respectively. In contrast, all patients who were intolerant to prior cBTKi remained on pirtobrutinib therapy for >24 cycles; median PFS 17 months vs unreached **(Figure 2A)**. Second, time-to-progression was shorter for patients with *BTK* mutation; 39% and 70% patients had PD at 12 and 18-months, respectively **(Figure 2B)**. In contrast, among 10 patients with wildtype-*BTK*, only 10% had PD at 18 months: median PFS 17 months vs unreached. At 12 and 18 months, for other poor-prognosis features, 30% and 60% disease progression occurred for complex karyotype, 33% and 83% for bulky lymph nodes **(Figure 2C-2D)** and 25% and 44% for unmutated *IGHV* (not shown). *TP53/*17pdel, *NOTCH1*, *ATM*/11qdel, 13qdel and trisomy 12 mutations showed similar median PFS compared to their WT cohorts, however, *XPO1* mutation was associated with a shorter PFS **(Supplemental Figure 2).**

***Pharmacological profiling of cells during pirtobrutinib therapy***

Data in **Figure 2** demonstrated that in most cases, PD occurred in first two years. Hence, we tested if cells taken around 10-20 cycles of pirtobrutinib when disease is still responsive and cells may be sensitive to pirtobrutinib were likely to undergo apoptosis when exposed to additional agents. To identify potential partners for pirtobrutinib, cells from 4 patients collected before and after 10-20 cycles of pirtobrutinib were treated *ex vivo* with additional targeted agents. In each case, percent of lymphocytes was similar in baseline and C10-20 pirtobrutinib samples. With each drug compared to pre-treatment sample, there was sensitization of cells after pirtobrutinib therapy **(Figure 3A-H)**. These data suggest that different targeted agents could be added or sequenced after several cycles of pirtobrutinib to enhance response.

Among the drugs, ibrutinib and venetoclax are heavily used for CLL and target BTK or Bcl-2 proteins which are critical for CLL cell survival. Hence, we compared cell death with these 2 agents alone and in combination. It is apparent that after a few cycles of pirtobrutinib, cells were generally more responsive to ibrutinib and venetoclax combination. Data from 4 patients clearly demonstrate sensitization of cells to combined ibrutinib and venetoclax after several cycles of pirtobrutinib **(Figure 3A-H)**.

***Disease progression is associated with loss of BTK inhibition by pirtobrutinib***

Among 14 patients with PD, plasma samples were available from 11 patients. These were tested for CCL3 **(Figure 4A)** and CCL4 **(Figure 4B)** levels to monitor suppression and progression of disease. Within a week of pirtobrutinib therapy, there was decline in the levels of both these chemokines which generally remained down throughout the 3 cycles of therapy. However, at PD there was marked, although heterogenous, increase in the levels of these chemokines suggesting activation of BCR signaling pathway. We analyzed these chemokine levels in patients who are still on pirtobrutinib therapy and maintaining clinical response. In general, baseline levels of CCL3 and CCL4 were lower in these patients. Also, in contrast to patients who had disease progression, for five patients who are still on pirtobrutinib therapy, both CCL3 and CCL4 levels decreased and remained low **(Figure 4C & D).**

***Disease progression is associated with enhanced sensitivity to ibrutinib.***

We then incubated peripheral blood cells *ex vivo* from 3 patients at time of disease progression and from 2 patients still on therapy with six different concentration (range 0.1 nM to 10 µM) of pirtobrutinib. Cells from patients on therapy were sensitive ($IC_{50}$, 532 nM) to pirtobrutinib compared to those from patients who had PD ($IC_{50}$, 6358 nM). Thus, *ex vivo* response to pirtobrutinib mirrored clinical disease progression on pirtobrutinib **(Figure 5A)**. Cells at time of progression were also incubated with six different concentrations of ibrutinib. Compared to pirtobrutinib, cells at PD were 10-fold more sensitive to ibrutinib ($IC_{50}$, 660 nM) **(Figure 5B)**. Data from six patients at time of PD suggested time-dependent resistance to pirtobrutinib while sensitivity to ibrutinib **(Figure 5C)**. In concert to viability data with ibrutinib or pirtobrutinib, BCR pathway inhibition was more pronounced with *ex vivo* ibrutinib compared to that with pirtobrutinib in 3 patients with PD. Collectively, these data suggest development of resistance to pirtobrutinib yet re-sensitivity to ibrutinib at time of PD on pirtobrutinib **(Figure 5D)**.

Baseline *BTK* status and associated cytogenetics are provided **(Supplemental Figure 3 A-B).** At time of PD, the C481 mutations decline with an increase in non-C481 *BTK* mutations. Acquired mutations were more frequent in patients with prior *BTK* mutations and had faster DP **(Supplemental Figure 3 C-E).** We postulate that these changes in *BTK* mutations at time of PD, may explain, at least partly, re-sensitization to ibrutinib.

***Disease progression was associated with reactivation of the BCR pathway and upregulation of MCL-1 and BCL-2 proteins.***

We postulated that our observations of increase in CCL3 and CCL4 and *ex vivo* resistance to pirtobrutinib at time of disease progression may represent activated signaling of the BCR pathway. To test this, immunoblots were analyzed for BCR-pathway proteins in 2 patients on therapy who harbored wildtype-*BTK* and 2 patients who had *BTK* mutation at time of progression **(Figure 6)**. In patients on therapy and with wildtype *BTK*, BCR pathway proteins were suppressed **(Figure 6A)**. AKT, however, showed increased phosphorylation at later time points. For patients with PD and accompanied second site *BTK* mutations, compared to baseline sample, there was an increase in pBTK and pNFkB in samples taken at progression and post-progression **(Figure 6B).**

Since we observed activation of NFkB protein in patients who had PD with *BTK* mutation, we analyzed changes in Bcl-2 family proteins in these samples. Interestingly, patients who are still on therapy, there was a decline in the level of total Bcl-2 and Mcl-1**(Figure 6A, lower panel)**. While in patients with PD and with non-C481 *BTK* mutations, there was an upregulation of Bcl-2 and Mcl-1 protein levels at time of progression **(Figure 6B, lower panel)**. The increase in Mcl-1 protein may represent BCR pathway reactivation. It is uncertain whether this increased Mcl-1 and Bcl-2 drive resistance to pirtobrutinib or not.

***Pharmacological profiling of CLL cells at progression on pirtobrutinib***

To explore therapeutic options available for pirtobrutinib-resistant CLL, PBMCs were *ex vivo* tested at time of progression. Blood samples from eleven patients at the time of PD were available for *ex vivo* incubation and pharmacologic profiling. These samples had high WBC count with 70 to 99% lymphocyte counts. Cells were incubated with 15 different conditions for 3 days, these included APR-246, venetoclax, AZD5991, ibrutinib, pirtobrutinib, and trametinib alone or either as doublets or triplets. These drugs were selected for their utility in CLL, targets in the disease, or their clinical availability. Apoptosis was measured every day for 3 days and numbers were normalized to vehicle (DMSO) control. Data suggest that there is heterogeneity regarding sensitivity to single agent. Venetoclax revealed a promising effective single agent as compared to ibrutinib and AZD5991. However, with double and triple drug combinations 70% to 90% cell death was observed in all cases **(Figure 7A-D)**. Extent of apoptosis was highly independent of type of 2$^{nd}$ site *BTK* mutation. In contrast to ibrutinib, the combination of pirtobrutinib with another drug, particularly venetoclax and APR-246, generally induced apoptosis from the second drug, further demonstrating resistance to pirtobrutinib **(Supplemental Figure 4A-B).** Cells from two patients with Bcl-2 mutations were more resistant to apoptosis (**Supplemental Figures 4C).**

Similar *ex vivo* data were obtained in 11 patients at time of disease progression and were plotted in a heatmap to determine apoptotic response to these drugs under different genetic and genomic profile which included non-*BTK* and second site *BTK* aberrations at PD. Except for double mutation (*BCL-2* and *BTK*), cells from all patients showed high apoptosis with doublets. Patients with or without complex karyotype also had high apoptotic outcome with combined BTKi and Bcl-2i exposure **(Figure 7E)**. Different genetic/genomic baseline profiles did not impact extent of apoptosis when drugs were combined (**Supplemental Figure 5A)**. At strict p-value, for apoptosis, the presence of *BCL-2* mutation was unfavorable, while *XPO-1* mutation was more sensitive to ibrutinib **(Supplemental Figure 5B).**

To further dissect, therapeutic vulnerability at different landscape of BCR and Bcl-2 pathways, heatmaps were done for each patient **(Figure 7F)**. Samples obtained from patients with either *BTK* or *PLCG2* mutations responded to combination of ibrutinib and venetoclax. However, double refractory disease (presence of both *BCL2* and *BTK* mutation) still represents a high unmet need for novel therapeutic options, as the mutual presence of these mutations provided poor apoptosis response to venetoclax and ibrutinib alone and combined. Besides, those with prior cBTKi and venetoclax showed less ex-vivo apoptosis when compared to those who received only cBTKi **(Supplemental Figure 5C).**

## Discussion

At genomic, biochemical, clinical, and molecular levels, we addressed three distinct questions in the current study for patients with previously cBTKi treated CLL who then received pirtobrutinib therapy (BRUIN trial).

First, a few features appeared to distinguish patients who progress within 24 cycles versus those who have PFS of >24 cycles on pirtobrutinib. I., patients who progressed on prior cBTKi appeared to have higher chance of PD as compared to those who were intolerant to prior cBTKi. Majority of patients in intolerant cohort did not have *BTK* mutations underscoring importance of wildtype versus mutant *BTK* and disease progression on pirtobrutinib. Previous report suggested similar PFS in patients with CLL, however, they compared only C481 mutations while we considered any BTK aberration. II., PD on pirtobrutinib was associated with complex karyotype. It is important to identify that among 10 patients with complex karyotype, 4 had both *TP53* mutations and del17p and additional 3 had only *TP53* mutations. III., there are more patients with baseline *BTK* mutations in the PD cohort, suggesting that while these patients initially respond to pirtobrutinib, within 24 cycles of pirtobrutinib, many of them do develop non-C481 *BTK* mutations resulting in progression. In our study, all patients with baseline *BTK* mutations were refractory to prior cBTKi and had a slightly higher risk of acquiring new second site *BTK* mutations as compared to the patients harboring wildtype *BTK* , which may partially explain the higher risk for mutant *BTK* to progress as compared to the wildtype cohort. Thus, we can also postulate that patients who have baseline *BTK* aberrations or complex karyotype have mutator phenotype and are prone to have additional mutations. IV., more CLL patients with wildtype *BTK*/ *PLCG2* remained in remission for a longer time. These observations underscore a need to evaluate pirtobrutinib in patients who have been treated before but are BTKi-naive. V., there were 3 patients who had mutated-*IGHV* gene and all three remain on therapy which is consistent with recent report where unmutated *IGHV* showed shorter PFS (26). With chemoimmunotherapy, it was shown that patients with mutated *IGHV* have better prognosis (33-35) on the other hand, single agent cBTKi suggest similar responses and PFS in CLL with mutated or unmutated *IGHV* (34,35). VI., a unique observation was that all 5 patients with *XPO1* mutations were in the PD group. This needs to be confirmed in full BRUIN CLL cohort. *XPO1* (E571) mutations are associated with neoplastic features by acquisition of additional aberrations in CLL (36) and promoted B-cell oncogenesis (37) substantiating our observation. Other high risk CLL features including *TP53* aberrations (17pdel and *TP53* mutation), *ATM*/11q del or *NOTCH1* mutations were present in both subgroups. Some of these prognostic factors (progression on prior cBTKi, baseline *BTK* mutations, bulky lymph node, complex karyotype) correlated with shorter time-to PD and shorter PFS; however, these features are mostly seen in previously treated CLL, suggesting that longer duration of responses may be observed with pirtobrutinib in the treatment-naïve setting.

Second component of current study was to determine if persistent CLL cells during 10-20 cycles of pirtobrutinib are still sensitive to pirtobrutinib and other targeted agents. Based on prior experience with ibrutinib, acalabrutinib, and zanubrutinib, most responders achieve partial remission to single agent cBTKi; complete remissions are rare and undetectable measurable residual disease (uMRD) status is generally not achieved. BRUIN trial data with pirtobrutinib monotherapy are in concordance with cBTKi observations. These results underscore a need for pirtobrutinib to be followed by other agent in sequence as consolidation or intensify by combination to achieve a deeper response, to achieve uMRD status.  For this purpose, we used pirtobrutinib-sensitive cells obtained during therapy for *ex vivo* incubations.  Interestingly, when tested prior to pirtobrutinib and during 10-15 cycles of pirtobrutinib, data clearly demonstrated that pirtobrutinib-treated cells have better apoptotic response to many targeted agents.  Pharmacological profiling of PBMCs at 10-15 cycles showed increased sensitivity to cBTKi such as ibrutinib, Bcl-2 antagonist like venetoclax, Mcl-1 direct inhibitor, a tool compound AZD5991, glutathione depleting drug APR-246, and MAP kinase inhibitor such as trametinib.  Sensitivity to these agents was higher than that observed in cells obtained at baseline.  In particular, cells obtained during pirtobrutinib therapy were sensitive to ibrutinib, venetoclax, and the combination.  These data provide pharmacological rationale to sequence pirtobrutinib monotherapy followed by venetoclax and/or cBTKi consolidation or to intensify therapy by adding cBTKi or/and Bcl-2i after a few cycles of pirtobrutinib.  There are prior precedents for both these strategies.  Venetoclax consolidation for CLL treated with ibrutinib resulted in high uMRD (38).  Intensification therapy  for combined ibrutinib and venetoclax (3 cycles of  ibrutinib followed by time-limited combined ibrutinib and venetoclax) in investigator-initiated protocol (39,40), CAPTIVATE trial (41), and GLOW study (42) resulted in clinical success and achievement of uMRD status.

Mechanism of this sensitization by pirtobrutinib was not evaluated, however, a few observations are in concert with the data.   C481S clone was highly susceptible to pirtobrutinib and was either eliminated or dramatically reduced after a few cycles of pirtobrutinib (24,43)  This may make population of remaining peripheral blood cells more sensitive to cBTKi such as ibrutinib.  Almost all patients on pirtobrutinib achieved partial remission after 8 to 12 cycles with high percent of persistent lymphocytes in the peripheral blood **(Figure 3 A-J)**.  Although we did not explore how pirtobrutinib treatment may enhance sensitivity to venetoclax, it was reported that BTK inhibition increases dependency on Bcl-2 (44) and decreases Mcl-1 protein level (45) which may further explain increased sensitivity to venetoclax following pirtobrutinib treatment.

Third focus, and perhaps the most significant question that was addressed in our study was to determine if cells at time of disease progression showed BCR pathway activation and if there are targeted agents that these pirtobrutinib-resistant cells would respond to.  This is an unmet need and from the BRUIN clinical trial (26) data, it is apparent that while pirtobrutinib is very effective in CLL with an overall response rate of 72% and median PFS of 19.6 months in CLL patients, yet after 12-18 months, 31 - 44% patients had disease progression.  We addressed if there are molecular rationales why pirtobrutinib relapsed cells should be treated with specific targeted agents.  Next-generation-sequencing suggested decrease or elimination of C481S clone after pirtobrutinib therapy (43).  Consistent with this observation, most patient samples show increased cell death with ibrutinib alone or combined with venetoclax.  Ibrutinib, but not pirtobrutinib, was able to inhibit phospho BTK at time of pirtobrutinib PD which is consistent with low $IC_{50}$ values of ibrutinib to several non-C481 *BTK* mutants (46).  We observed upregulation of Bcl-2 and Mcl-1 proteins in samples at time-of-progression.  Bcl-2 is successfully targeted by venetoclax, and Mcl-1 is neutralized by many agents that directly bind including AZD5991(47). We tested ibrutinib as well to explore if cells can be re-sensitized to ibrutinib. The

MEK/ERK inhibitors was tested as it can intensify the effect of BTKi and also target the activated ERK pathway at time of PD. In concert, cells at time of PD showed sensitivity to venetoclax and AZD5991 alone or combined with ibrutinib. Immunoblot data further suggested activation of ERK at time of PD. Trametinib, an inhibitor of MEK pathway (48), was effective in inducing cell death. Importantly, other genomic signatures (*TP53* and *ATM*) and complex karyotype did not impact sensitivity of cells to targeted agents. E571 *XPO1* mutations in CLL cells confer ibrutinib sensitivity as reported before in lymphomas (49). Patients with *BCL-2* mutations, however, showed relative resistance which is consistent with the observation that those with prior history of BTKi + Bcl-2i showed shorter PFS (26). In our data, those with prior BTKi and venetoclax showed less apoptosis *ex vivo* as compared to those treated with prior BTKi only. Clinically post pirtobrutinib patients were retreated with cBTKi and venetoclax with or without obinutuzumab, PKC-b inhibitor, or CAR-T cell therapy **(Supplemental Table 3).** Ongoing clinical trials that combine pirtobrutinib and venetoclax with obinutuzumab (NCT05536349) or with rituximab (NCT04965493) will further elucidate benefits of these combinations.

In conclusion, we discovered that patients with underlying *BTK* mutations, complex karyotype and bulky lymph nodes were more prone to CLL progression on pirtobrutinib. Our profiling data provide options for pirtobrutinib sequencing strategies for patients who are pirtobrutinib sensitive and combination intensification strategies especially for those who have resistance disease due to progression. Although our sample size is small, and done in one center, the data provide insights on factors that may influence longer response to pirtobrutinib following ibrutinib failure.

## Declarations

### Acknowledgements

We thank Dr. John Pagel, Loxo/Lilly Oncology for reviewing this manuscript and providing critical and valuable comments. This work was funded partly by the MD Anderson CLL Moon Shot™ program and by Loxo Oncology. Core facilities were utilized for this project and are funded by MD Anderson Cancer Center Support Grant, P30 CA016672, from the National Institutes of Health. Authors thank Stephanie Gabriella Zelaya for coordinating sample transportation from patients' room to research laboratories.

### Authorship Contributions

**S.I.T.** performed all experiments in primary PBMCs obtained from patients on pirtobrutinib clinical trial, analyzed data, made figures and wrote portions of the manuscript. **B.A.** coordinated the collection of patient samples and reviewed portions of the manuscript. **G.M.** performed all statistical analyses of data, reviewed manuscript, and wrote statistical sections. **L.I.** processed most samples, isolated PBMCs for storage and distribution, and performed ELISA for chemokines. N.T. performed some of the experiments. N.S. performed some of the experiments, **N.J., A.F.,** and **P.T.** treated many patients and provided patient samples and data. **K.P.** was responsible for genomic sequencing and provided critical comments on the manuscript. **S.D.** provided patient characteristics and provided critical comments on the manuscript. **W.G.W.** is director of the clinical trial. He identified patients, provided samples from patients, participated in intellectual discussion, and reviewed manuscript. **V.G.** conceptualized the project, obtained funding, analyzed data, wrote majority of the manuscript and critically revised the manuscript.

#### Conflict of Interest Disclosures

Specifically, for the current investigations, V.G. received sponsored research agreement from Loxo Oncology (now a subsidiary of Lilly Oncology). Previously, for other investigations, V.G. received research funding from Pharmacyclics, Acerta, Gilead, Sunesis, Infinity, AbbVie, Clear Creek Bio. W.G.W. received research funding from GSK/Novartis, Abbvie, Genentech, Pharmacyclics LLC, AstraZeneca/Acerta Pharma, Gilead Sciences, Juno Therapeutics, KITE Pharma, Sunesis, Miragen, Oncternal Therapeutics, Inc., Cyclacel, Loxo Oncology, Inc., Janssen, Xencor. The remaining authors declare no competing financial interests.

## Tables

Table 1 is available in the Supplementary Files section.

## Figures

#### Figure 1.

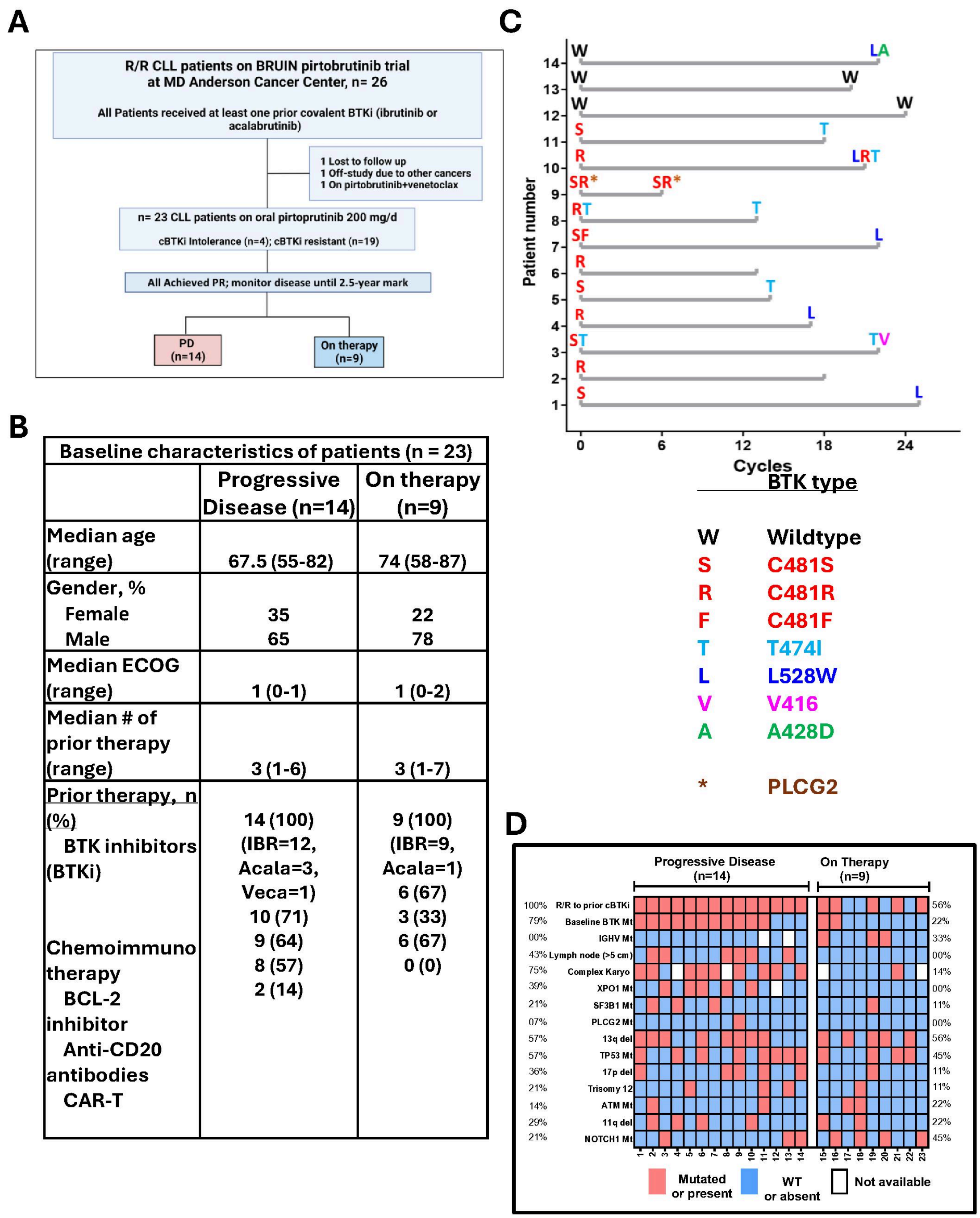

| Baseline characteristics of patients (n = 23) | | |
|---|---|---|
| | Progressive Disease (n=14) | On therapy (n=9) |
| Median age (range) | 67.5 (55-82) | 74 (58-87) |
| Gender, % | | |
| Female | 35 | 22 |
| Male | 65 | 78 |
| Median ECOG (range) | 1 (0-1) | 1 (0-2) |
| Median # of prior therapy (range) | 3 (1-6) | 3 (1-7) |
| Prior therapy, n (%) | | |
| BTK inhibitors (BTKi) | 14 (100) (IBR=12, Acala=3, Veca=1) | 9 (100) (IBR=9, Acala=1) |
| Chemoimmuno therapy | 10 (71) | 6 (67) |
| BCL-2 inhibitor | 9 (64) | 3 (33) |
| Anti-CD20 antibodies | 8 (57) | 6 (67) |
| CAR-T | 2 (14) | 0 (0) |

Figure 1

**Baseline features of CLL patients who either progressed on pirtobrutinib or remained on therapy beyond 30 cycles of pirtobrutinib.**

**(A).** Consort diagram to provide patient distribution. From the BRUIN trial, at MD Anderson 26 patients were enrolled on pirtobrutinib clinical trial. Three patients were taken off before completing 2.5 years of therapy/or before experiencing disease progression for indicated reasons. Out of the 23 patients who completed at least 30 cycles of therapy, we had 14 patients who had PD (including one patient with Richter transformation) while 9 patients still remain on therapy beyond 24 cycles of pirtobrutinib. **(B).**Baseline features between those who progressed and those on therapy (Rx) are provided. Data were obtained from patients' clinical records. No statistical significance in baseline features between the two cohorts was noticed **(C).** Swimmer's plot for 14 patients who had disease progression by 30 cycles of pirtobrutinib. Baseline BTK mutational status is indicated for each patient at cycle 0. All patients achieved PR on pirtobrutinib. Patient 12 had Richter's transformation while other 13 had CLL progression. BTK mutational status at time of progression is indicated at the respective cycle when disease was progressed. One patient had PLCG2 mutation (indicated with an *). **(D).** OncoPrint data showing the most common baseline features associated with PD. Data were obtained from patients' medical records, cytogenetics, and targeted-NGS analyses. Fishers exact test was used to determine association between disease status and biomarkers of interest. P-values obtained after multiple tests were adjusted using Benjamini-Hochberg (BH) method. Disease progression was significantly associated with prior BTKi r/r (p=0.03), baseline mutant BTK (p=0.03), complex karyotype (p=0.03), bulky (>5cm) lymph node (p= 0.05), and IGVH unmutated status (p=0.05).

Figure 2.

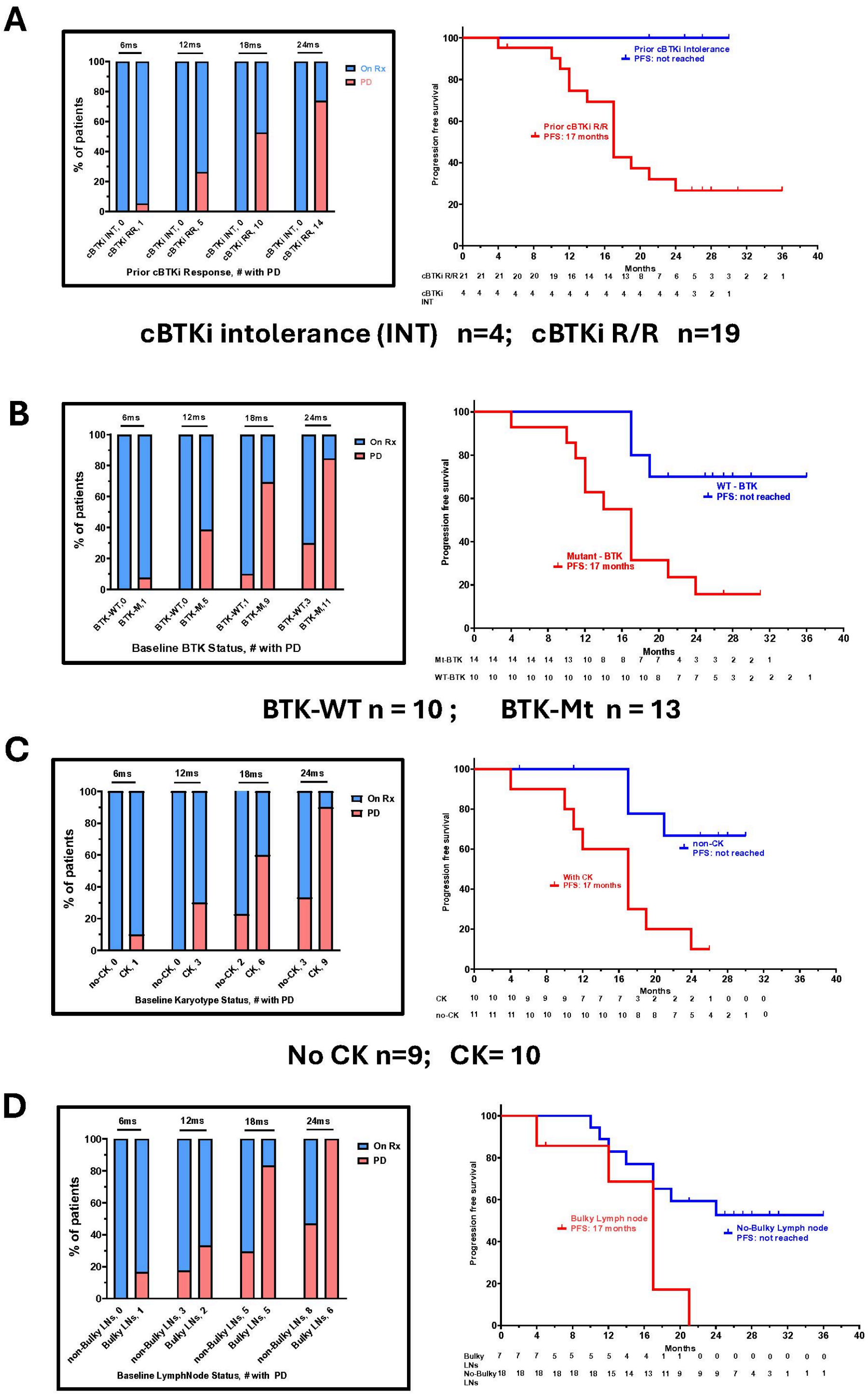

### Figure 2

#### Frequency and velocity of PD among CLL patients with different genomics and their impact on PFS.

**(A-D).** Bar-graph to show number of patients with PD within first two years at 6-month intervals. Numbers on the X-axis represent number of patients having PD. Kaplan-Meier curve for PFS is shown beside the bar graph. Shorter PFS was associated with history of resistance to prior cBTKi {hazard ratio (HR)=

4.4; 95% confidence interval (CI) (1.196 to 16.28)}, baseline mutant BTK {HR= 4.6; 95% CI (1.595 to 13.13)}, complex karyotype {HR= 6; 95% CI (1.706 to 21.03)}, as well as the presence of bulky lymph nodes {HR= 5.4; 95% CI 1.295 to 22.11)}. Relapsed/refractory (R/R) disease; WT, wildtype-*BTK*and Mt, mutant-*BTK* at baseline; CK, complex karyotype; LN lymph nodes.

**Figure 3.**

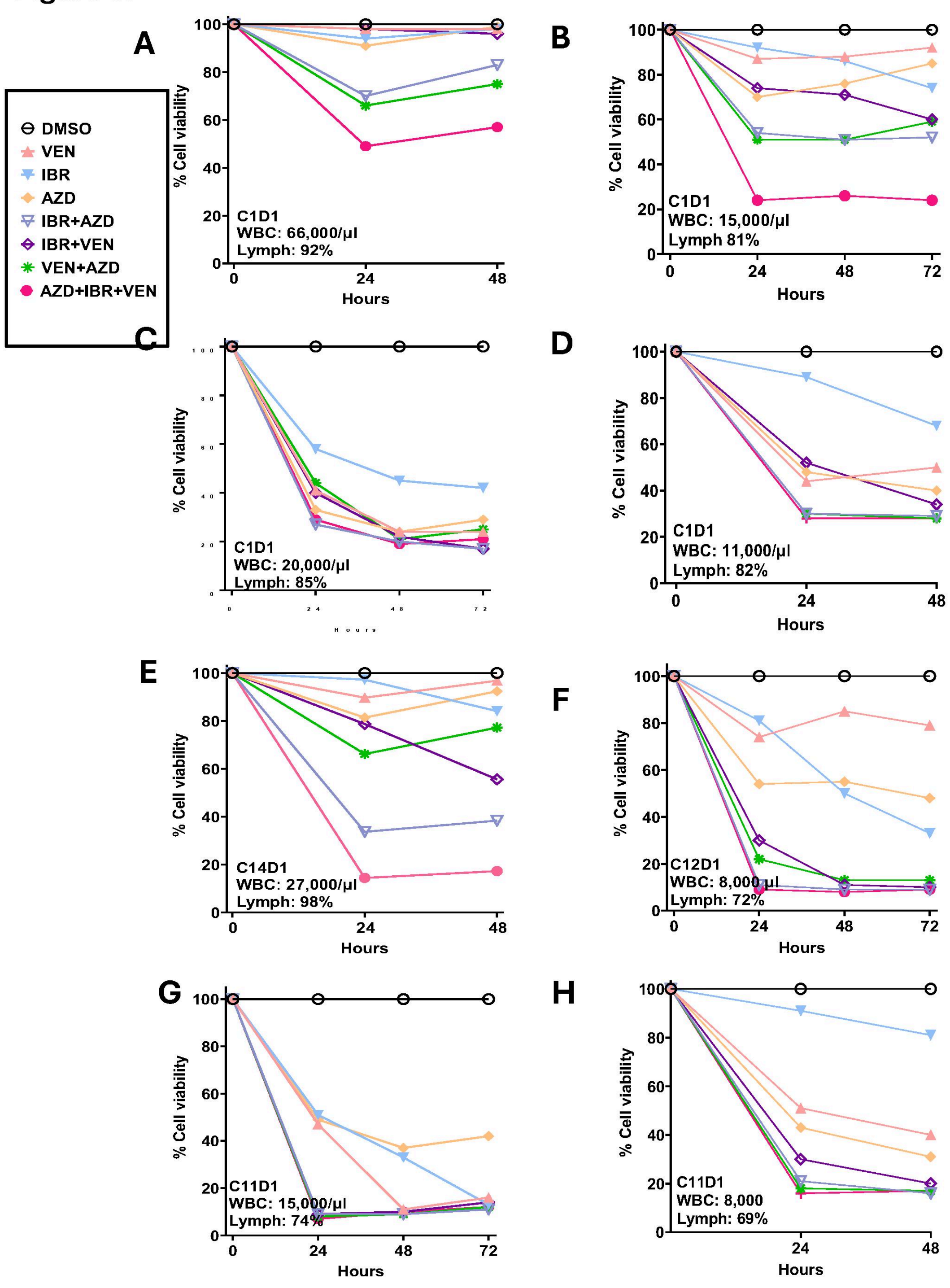

### Figure 3

**Sensitization of cells during pirtobrutinib therapy to venetoclax, AZD5991 and ibrutinib.**

**(A-H).** PBMCs were collected from four patients before start of pirtobrutinib **(A-D)** and same four patients after 10-20 cycles **(E-H)** of pirtobrutinib. Each upper panel represents a single patient and their respective post pirtobrutinib samples are in the lower panel. Total white blood cell (WBC) and lymphocyte % for each patient is provided in the lower left. Cells were incubated with AZD5991, venetoclax, ibrutinib, or their combinations. Viability was measured *ex vivo* at time 0 and at day 1, 2, and 3 using Annexin V-PI assay.C, cycle of pirtobrutinib; PT, patient; WBC, white blood cell; ALC, absolute lymphocyte count; VEN, venetoclax; IBR, ibrutinib; AZD, AZD5991.

**Figure 4.**

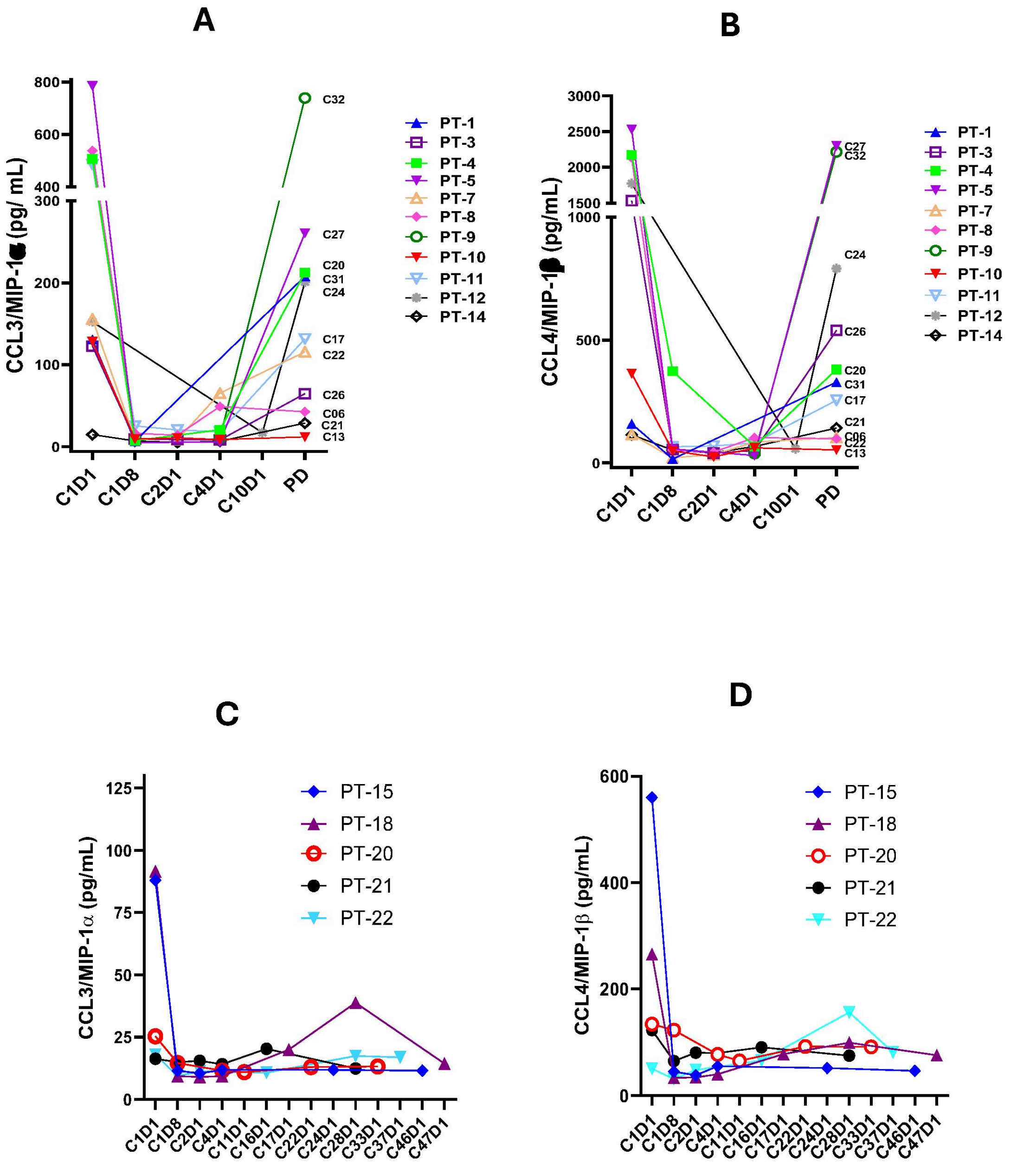

### Figure 4

#### Levels of CCL3 and CCL4 in plasma during pirtobrutinib therapy and at time of disease progression.

**(A-B).**Plasma levels of CCL3 **(A)** and CCL4 **(B)** during therapy and at time-of-progression from patients (n = 11) on pirtobrutinib therapy. **(C-D).** Plasma levels of CCL3 **(C)**and CCL4 **(D)** from patients (n = 5) who are on pirtobrutinib therapy for >28 cycles. Plasma was isolated from peripheral blood and analyzed by ELISA as indicated in the Methods section.

### Figure 5.

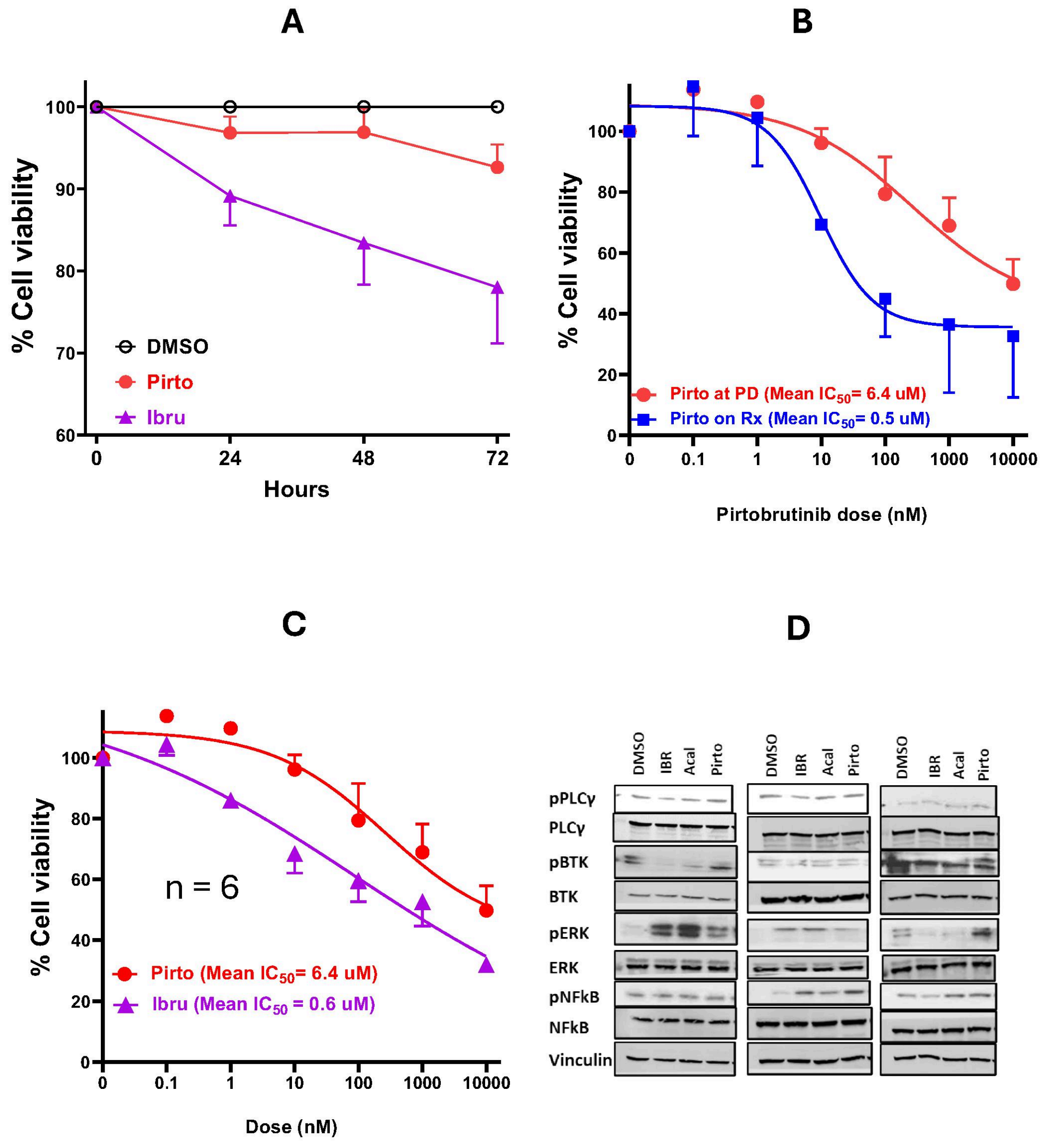

### Figure 5

**Sensitivity of PBMCs to ibrutinib at time of disease progression on pirtobrutinib therapy.**

**(A).** Resazurin cell viability assay to PBMCs during therapy (blue line, n = 2) or at time of PD (red line, n = 3) on pirtobrutinib. Cells were obtained from blood and were incubated with indicated concentrations of pirtobrutinib for 72 hours. **(B).** Resazurin cell viability assay to pirtobrutinib (red line, n = 3) or ibrutinib (purple line, n = 3) at time of PD on pirtobrutinib. Cells were obtained from blood at time-of-progression and were incubated with ibrutinib (purple line) or pirtobrutinib (red line) for 72 hours. **(C).** Time-dependent cytotoxicity to PBMCs during *ex vivo* ibrutinib (purple line, n = 6), or pirtobrutinib (red line n= 6). Cells were obtained from patients and were incubated with 10 μM pirtobrutinib or ibrutinib for one, two or three days. Cell death was measured by AnnexinV-PI flowcytometry. **(D)**. Immunoblot for patients'cells at time of disease progression and after ex-vivo incubation with different BTK inhibitors (1μM) for 24 hours. IBR, ibrutinib; Acal, acalabrutinib; Pirto, Pirtobrutinib.

**Figure 6.**

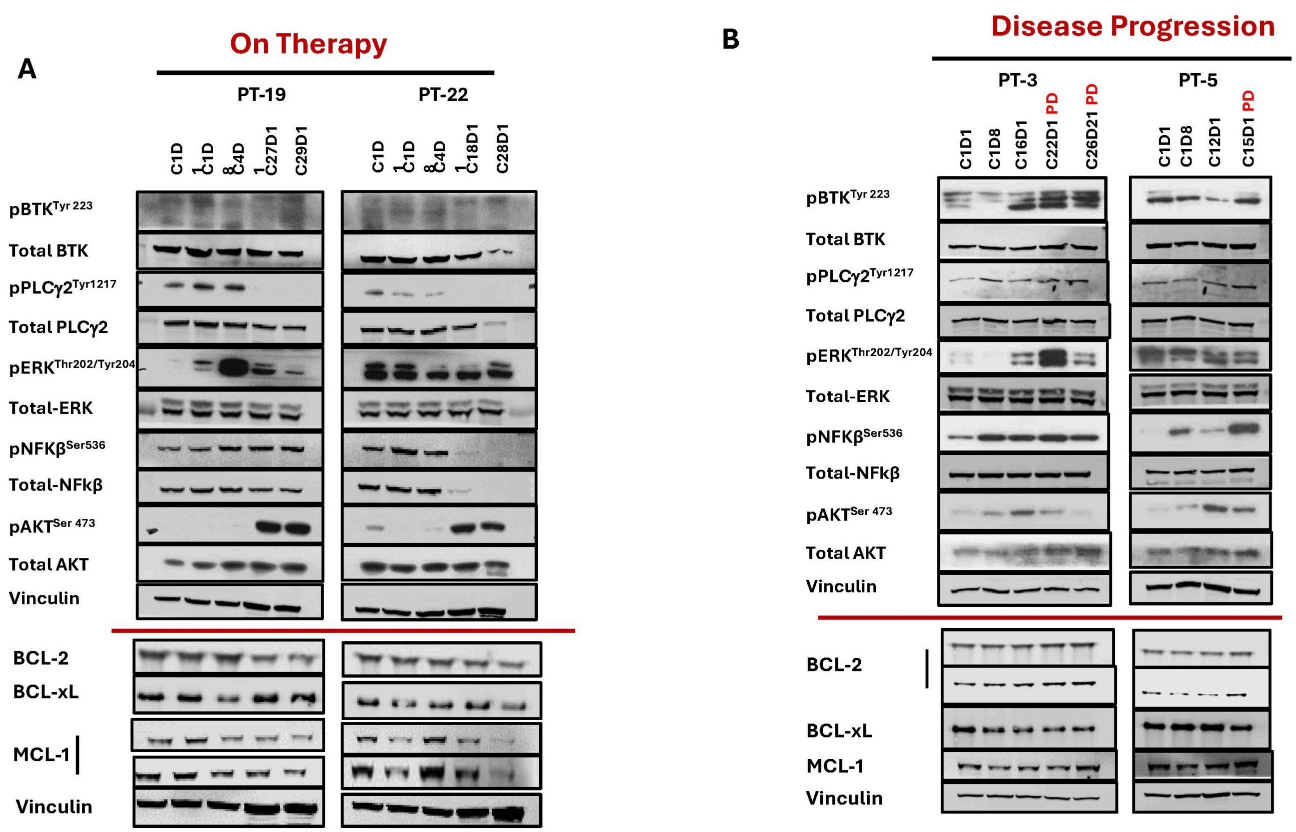

### Figure 6

**Suppression and activation of BCR pathway and Bcl-2 survival proteins during therapy and at time of PD.**

**(A-B).** Peripheral blood samples were obtained at indicated times during therapy from two patients and at time of progressive disease progression (PD) from two patients. All Patients who progressed had *BTK* mutations at baseline (n=2). Patients who are still on therapy had wildtype-*BTK* at baseline and did not have PD at >24 cycles (n=2). Top panels. Proteins were extracted and immunoblotted for total and phospho proteins in the BCR pathway (BTK, PLCγ2, ERK, NFkB, and AKT). Vinculin was used as a loading control. Bottom panels. Proteins were extracted and immunoblotted for Bcl-2 survival proteins including Bcl-2 (long and short exposure), Bcl-XL, Mcl-1 (long and short exposure). Vinculin was used as a loading control.

### Figure 7.

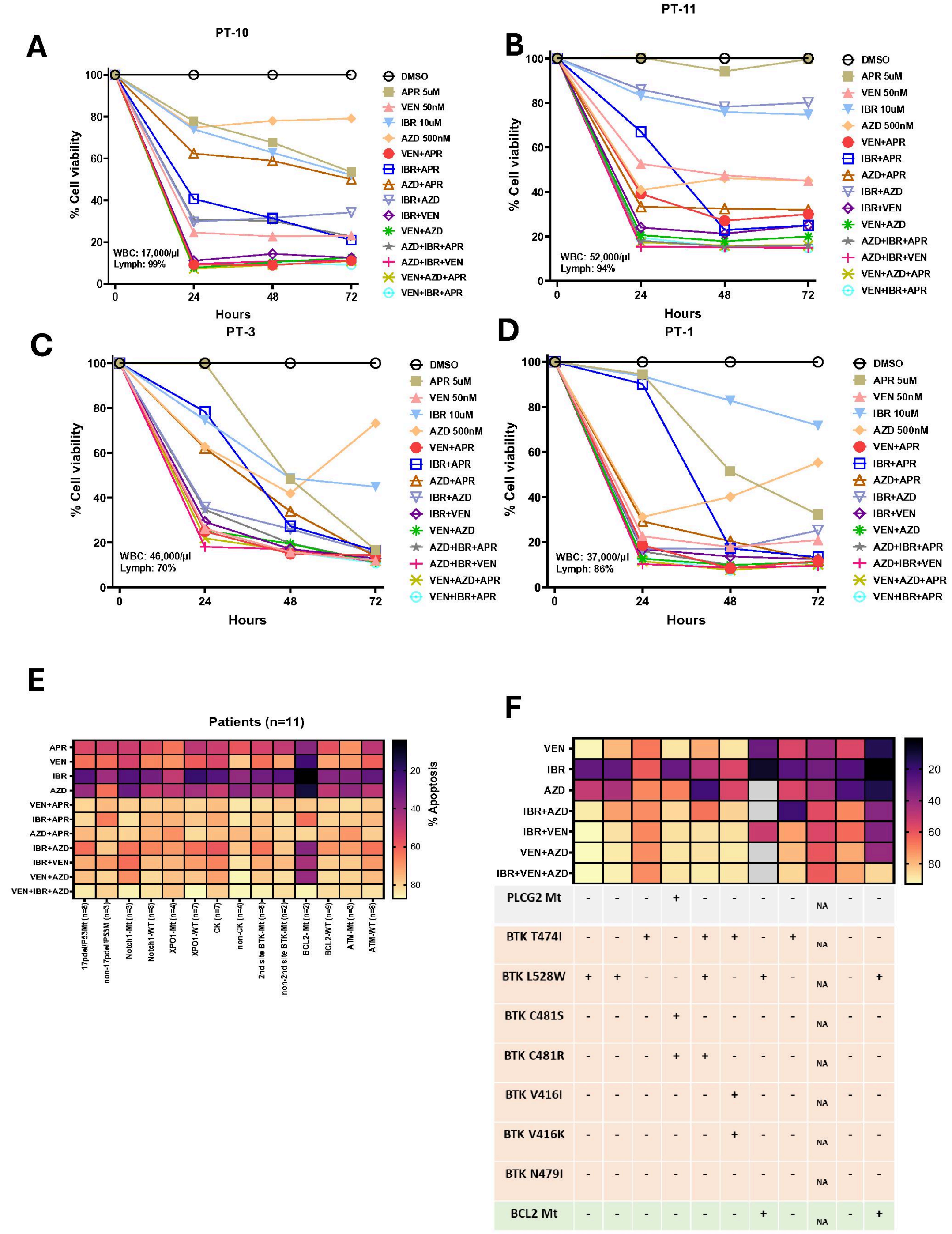

### Figure 7

#### Pharmacological profiling of cells at time of PD.

**(A-D).**PBMCs were collected from 4 patients at time of PD. The cells were incubated *ex vivo* with single agent ibrutinib, venetoclax, APR-246, AZD5991, and their double or triple combinations for up to three days. Viability was measured every day using Annexin-PI assay. Mutational status at PD were as below.

**(A).**PT-10. *BTK* (L528W 7%, C481R 12%, T474I 83%); *BIRC3*(79%); *XPO1* (42%); *DNMT3TA* (5%); *TP53* (Two mut, 40% and 5%); *ZRSR2*(5%). **(B).** PT-11. *BTK* (T474I, 28%); *DNMT3TA*(5%); *HVCN1* (27%); *KMT2D* (14%); *MED12* (90%); *TP53*(79%). **(C).** PT-3. *BTK* (T474I 73%, V416K 5%, V416I 5%); *NOTCH1*(36%); *SPEN* (36%); *XPO1* (5%); *IRF4* (5%). **(D).** PT-1. *BTK* (L528W, 78%); *TP53* (65%); *ZMYM3* (80%); *MAP2K1*(39%). **(E)**. Heat map showing % apoptosis during *ex vivo* incubations in samples obtained at time of PD in multiple genomic background. PBMCs were taken at time of PD and were incubated with APR-246, venetoclax, ibrutinib, and AZD5991 and their combinations for three days. **(F)**. Heat map showing % apoptosis of cells at time of PD in multiple *BTK/PLCG2* mutations. PBMCs were taken at time of PD and were incubated with venetoclax, ibrutinib, and AZD5991 and their combinations. Genomic background was determined using next generation sequencing of PBMCs at time of PD. Cell death was measured using Annexin V-PI assay. C, cycle of pirtobrutinib; PT, patient; WBC, white blood cell; Lymph, percentage of lymphocyte; APR, APR-246; VEN, venetoclax; IBR, ibrutinib; AZD, AZD5991.

## Supplementary Files

This is a list of supplementary files associated with this preprint. Click to download.

- SupplementalTable1FINAL.pdf
- SupplementalTable2FINAL.pdf
- SupplementalTable3FINAL.pdf
- Table1V5FINAL.pdf
- SupplementalFigureLegendsV4FINAL.docx
- SupplementalFiguresV9FINAL.pdf
